# *Salmonella* Combination Vaccines: Moving Beyond Typhoid

**DOI:** 10.1093/ofid/ofad041

**Published:** 2023-06-02

**Authors:** Calman A MacLennan, Jeffrey Stanaway, Stephanie Grow, Kirsten Vannice, A Duncan Steele

**Affiliations:** Enteric and Diarrheal Diseases, Global Health, Bill & Melinda Gates Foundation, Seattle, Washington, USA; Jenner Institute, University of Oxford, Oxford, United Kingdom; Institute for Health Metrics and Evaluation, University of Washington, Seattle, Washington, USA; Enteric and Diarrheal Diseases, Global Health, Bill & Melinda Gates Foundation, Seattle, Washington, USA; Enteric and Diarrheal Diseases, Global Health, Bill & Melinda Gates Foundation, Seattle, Washington, USA; Enteric and Diarrheal Diseases, Global Health, Bill & Melinda Gates Foundation, Seattle, Washington, USA

**Keywords:** nontyphoidal, paratyphoid, *Salmonella*, typhoid, vaccines

## Abstract

There is now a robust pipeline of licensed and World Health Organization (WHO)–prequalified typhoid conjugate vaccines with a steady progression of national introductions. However, typhoid fever is responsible for less than half the total global burden of *Salmonella* disease, and even less among children aged <5 years. Invasive nontyphoidal *Salmonella* disease is the dominant clinical presentation of *Salmonella* in Africa, and over a quarter of enteric fever in Asia is due to paratyphoid A. In this article, we explore the case for combination *Salmonella* vaccines, review the current pipeline of these vaccines, and discuss key considerations for their development, including geographies of use, age of administration, and pathways to licensure. While a trivalent typhoid/nontyphoidal *Salmonella* vaccine is attractive for Africa, and a bivalent enteric fever vaccine for Asia, a quadrivalent vaccine covering the 4 main disease-causing serovars of *Salmonella enterica* would provide a single vaccine option for global *Salmonella* coverage.

## Teaser

Typhoid conjugate vaccines are efficacious against *Salmonella* Typhi. Globally, most *Salmonella* disease in humans is caused by non-Typhi *Salmonella* serovars. Nontyphoidal *Salmonella* and paratyphoid require combination *Salmonella* vaccines. A quadrivalent *Salmonella* vaccine could prevent a large majority of *Salmonella* disease.

**Figure 1.**
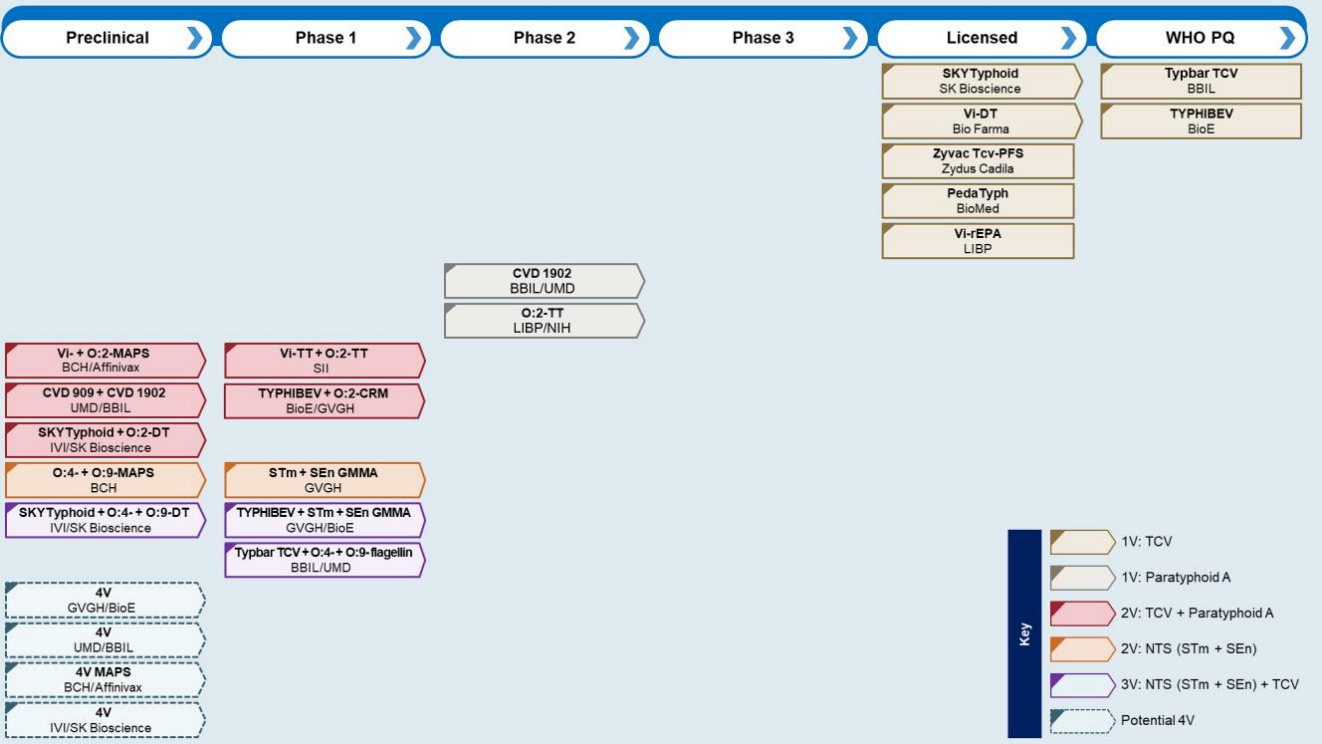
*Salmonella* combination vaccine pipeline including licensed and World Health Organization–prequalified typhoid conjugate vaccines, monovalent paratyphoid A vaccines in clinical development, and potential quadrivalent *Salmonella* vaccines. Arrowed boxes indicate ongoing development. Abbreviations: 1V, monovalent; 2V, bivalent; 3V, trivalent; 4V, quadrivalent; BBIL, Bharat Biotech International Ltd; BCH, Boston Children's Hospital; BioE, Biological E; GMMA, generalized modules for membrane antigens; GVGH, GlaxoSmithKline Vaccines Institute for Global Health; IVI, International Vaccine Institute; LIBP, Lanzhou Institute of Biological Products; NIH, National Institutes of Health; NTS, nontyphoidal *Salmonella*; PQ, prequalified; SEn, *Salmonella* Enteritidis; STm, *Salmonella* Typhimurium; SII, Serum Institute of India; TCV, typhoid conjugate vaccine; UMD, University of Maryland; WHO, World Health Organization.

Over the past few years, there has been considerable success in development of conjugate vaccines to tackle the global problem of typhoid fever, a disease endemic in many low- and middle-income countries (LMICs) [1–3] and driver of antimicrobial resistance (AMR) [4–7]. In 2017, a first typhoid conjugate vaccine (TCV), Vi-TT (Typbar TCV, Bharat Biotech International Ltd, India) [8], was prequalified by the World Health Organization (WHO) [9] (Figure 1). This vaccine consists of the Vi capsular polysaccharide of *Salmonella enterica* serovar Typhi conjugated to tetanus toxoid (TT). Prior to this, the vaccine had been licensed by the Drugs Controller General of India (DCGI) in 2013, for use down to 6 months of age, enabling its use in the private market in India.

Prequalification followed the demonstration of efficacy in a controlled human infection model (CHIM) at the University of Oxford [10], opening the way for procurement by Gavi, the Vaccine Alliance, and the United Nations Children’s Fund (UNICEF) [11], and national introductions of Typbar TCV in Pakistan, Liberia, and Zimbabwe [12–14]. Postlicensure studies of Typbar TCV in Nepal, Bangladesh, and Malawi, coordinated by the Typhoid Vaccine Acceleration Consortium (TyVAC) [15], have demonstrated efficacies of 82% [16], 85% [17], and 84% [18], respectively, in the 12–18 months following 1 dose. Separately, introduction of Typbar TCV in Pakistan, driven by an outbreak of extensively drug-resistant (XDR) typhoid [19, 20], was shown to have 95% effectiveness against all typhoid fever and 97% effectiveness against XDR typhoid fever [21]. Antibody persistence studies indicate that protection conferred by this vaccine may extend for years [22].

In addition to Typbar TCV, a second TCV, Vi-CRM_197_ (TYPHIBEV, Biological E, India), consisting of Vi conjugated to the nontoxic mutant of diphtheria toxin [23], was licensed by the DCGI and prequalified by the WHO in 2020 [2]. Vi-CRM_197_ had its first national introduction in Nepal in 2022 [24]. Also in 2022, a Vi-DT vaccine of Vi conjugated to diphtheria toxoid (DT), SKYTyphoid, developed by SK Bioscience (South Korea) [25], received an export license from the Korean Ministry of Food and Drug Safety, and another Vi-DT vaccine developed by Bio Farma (Indonesia) has recently been licensed by the Indonesian Food and Drug Authority.

Two other Vi-TT TCVs have been licensed in India: PedaTyph (BioMed, India) in 2008 [26] and ZyvacTcv-PFS (Zydus Lifesciences, India) in 2017 [1]. In addition, a TCV consisting of Vi bound to nontoxic recombinant exoprotein A of *Pseudomonas aeruginosa* (Vi-rEPA) TCV was developed by the Lanzhou Institute of Biological Products (LIBP, China) and licensed for use in China [27]. This is similar to the prototype TCV developed by Robbins and colleagues at the US National Institutes of Health, which was first shown to have efficacy among children in a phase 3 trial in Vietnam in 2001 [28].

## GLOBAL BURDEN OF *SALMONELLA* DISEASE

**Figure 2.**
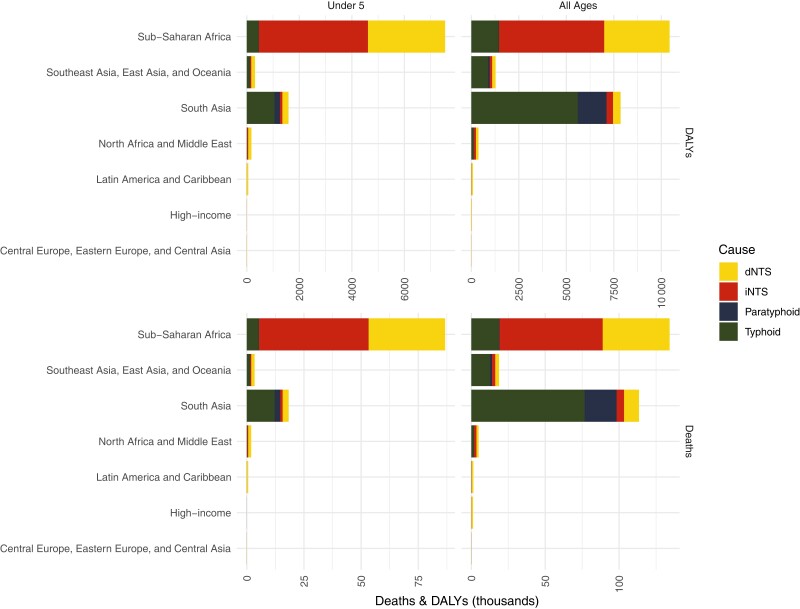
Deaths and disability-adjusted life-years attributable to *Salmonella enterica* in children aged <5 years and all ages by disease type and geographic region. From Global Burden of Disease 2019 enteric and diarrheal disease burden estimates [30, 31]. Abbreviations: DALYs, disability-adjusted life-years; dNTS, diarrheal nontyphoidal *Salmonella* disease; iNTS, invasive nontyphoidal *Salmonella* disease.

While typhoid fever, otherwise known simply as “typhoid,” which is caused by *S.* Typhi, is the commonest form of bacterial bloodstream infection in South and Southeast Asia [29], the global burden of *Salmonella* extends well beyond this disease (Figure 2). In Africa, particularly sub-Saharan Africa, invasive nontyphoidal *Salmonella* (iNTS) disease, predominantly caused by the nontyphoidal *Salmonella* (NTS) serovars *S.* Typhimurium and *S.* Enteritidis, is the main cause of bacterial bloodstream infections, and has high associated case fatality rates of 15% [32] and high levels of AMR [33]. The same 2 serovars, *S.* Typhimurium and *S.* Enteritidis, are also responsible for a large burden of diarrheagenic NTS (dNTS) disease globally. In South and Southeast Asia, *S.* Paratyphi A causes paratyphoid A fever, which is clinically indistinguishable from typhoid fever. Typhoid and paratyphoid fever are known collectively as enteric fever. Paratyphoid A accounts for over a quarter of cases of enteric fever [30] and is also responsible for an important burden of AMR [7].

Global Burden of Disease (GBD) 2019 estimates from the Institute of Health Metrics and Evaluation attribute 40% of all *Salmonella* deaths to typhoid fever, but only 17% of deaths in children aged <5 years. Other *Salmonella* deaths are divided between iNTS disease (29% total deaths, 45% deaths in children under 5), NTS diarrhea (22% total deaths, 36% deaths in children under 5; though note uncertainty here with wide 95% confidence intervals), and paratyphoid fever (9% total deaths, 2% deaths in children under 5) [30, 31] (Table 1).

**Table 1.** Global Burden of Disease 2019 Enteric and Diarrheal Disease Burden Estimates in Children <5 Years of Age and All Ages

Pathogen/Disease	Children Aged <5 y		All Ages	
	Deaths	DALYs	Deaths	DALYs
Rotavirus	151 514 (70 588–266 416)	13 568 166 (6 391 731–23 612 454)	235 331 (110 221–415 457)	17 071 346 (8 567 481–29 151 299)
*Salmonella enterica* (combined)^a^	111 023	9 689 494	274 059	20 075 278
*Shigella*	93 831 (35 860–185 931)	8 402 887 (3 274 243–16 542 456)	148 202 (61 975–284 541)	10 602 910 (4 538 791–20 242 702)
Adenovirus	83 492 (43 914–143 867)	7 415 744 (3 914 145–12 770 032)	107 065 (63 519–172 993)	8 321 445 (4 701 161–14 131 064)
*Cryptosporidium*	77 523 (15 962–190 426)	6 862 766 (1 463 118–16 773 435)	133 423 (26 424–360 303)	8 170 908 (1 797 798–20 226 898)
*Campylobacter*	58 911 (24 006–116 236)	5 324 624 (2 230 951–10 387 448)	139 080 (47 005–304 635)	7 307 840 (3 204 436–14 174 900)
Cholera	55 701 (28 044–93 931)	4 837 150 (2 438 859–8 153 152)	117 241 (71 090–177 806)	7 134 552 (4 032 717–11 139 174)
iNTS^a^	49 869 (27 161–80 009)	4 318 828 (2 355 108–6 931 248)	79 046 (43 013–124 207)	6 114 292 (3 323 425–9 705 739)
Norovirus	43 481 (11 754–99 172)	3 962 128 (1 192 131–8 842 284)	135 798 (25 103–303 735)	6 879 357 (2 085 136–14 198 132)
dNTS^a^	39 493 (4376–107 810)	3 500 124 (426 309–9 395 645)	61 647 (4376–190 566)	4 269 216 (475 319–12 056 915)
*Aeromonas*	19 651 (7871–39 046)	1 744 504 (709 751–3 449 201)	28 019 (12 945–50 322)	2 073 448 (932 430–3 883 407)
*Entamoeba*	19 049 (4952–50 300)	1 706 349 (448 942–4 485 968)	33 409 (10 529–82 410)	2 539 799 (850 865–6 186 972)
Typhoid fever^a^	18 934 (7228–38 033)	1 635 423 (625 745–3 279 949)	110 029 (52 810–191 206)	8 053 346 (3 864 905–13 925 252)
EPEC	15 844 (7447–29 987)	1 412 061 (667 888–2 658 679)	20 613 (10 118–37 221)	1 679 423 (858 148–3 045 518)
ETEC	12 399 (4983–26 372)	1 133 338 (466 320–2 389 033)	39 802 (18 039–76 964)	1 695 355 (828 589–3 252 268)
Paratyphoid fever^a^	2727 (844–6588)	235 120 (72 825–567 486)	23 337 (9801–45 680)	1 638 424 (682 263–3 206 062)
*Clostridioides difficile*	2102 (1306–3218)	182 179 (113 038–278 999)	32 134 (28 131–36 549)	870 814 (722 988–1 052 360)

Data are presented as No. (95% confidence interval).

Abbreviation: DALYs, disability-adjusted life-years; dNTS, nontyphoidal *Salmonella* diarrhea; EPEC, enteropathogenic *Escherichia coli*; ETEC, enterotoxigenic *Escherichia coli*; iNTS, invasive nontyphoidal *Salmonella*.

iNTS, dNTS, typhoid fever, and paratyphoid fever contribute to *Salmonella enterica* (combined) burden.

## CONCEPT OF A COMBINATION *SALMONELLA* VACCINE

Despite the different disease presentations attributable to *Salmonella*, they are all caused by the same species of bacteria, *Salmonella enterica*, albeit by 4 different serovars, as discussed above. According to GBD 2019 estimates, a vaccine that covers these 4 serovars of *S enterica* could prevent up to approximately 274 000 total deaths (111 000 deaths in children under 5) and approximately 20 million total disability-adjusted life-years (DALYs; 9.7 million among children under 5) (Table 1).

The potential impact of a combination *Salmonella* vaccine is indicated by the higher numbers of avertable deaths and DALYs in all ages for such a vaccine compared with vaccines for the other major etiologies of enteric and diarrheal diseases including rotavirus and *Shigella* (Table 1). Extrapolating from GBD 2019 estimates, *Salmonella* deaths account for 19% of all enteric and diarrheal disease deaths, and 8% of enteric and diarrheal disease deaths in children aged <5 years. This compares with rotavirus (16% of all diarrhea deaths and 10% in children under 5), *Shigella* (10% of all diarrhea deaths and 6% in children under 5), and cholera (8% of all diarrhea deaths and 4% in children under 5).

In addition to the high global burden of disease attributable to *Salmonella*, difficulties with diagnosing and treating life-threatening *Salmonella* infections provide further justification for the development of combination *Salmonella* vaccines. Presentation is often with fever alone, precluding diagnosis based on clinical finding. Laboratory diagnosis currently relies on either blood culture for *S.* Typhi and *S.* Paratyphi A and invasive nontyphoidal *Salmonella* disease or stool culture for diarrheagenic disease. The necessary infrastructure for such testing is rare in LMIC settings and, when available, usually takes 2 or more days, precluding this as a point-of-care diagnostic. Finally, growing levels of AMR mean that, increasingly, *Salmonella* infections cannot be treated successfully with available antibiotics, which supports the concept of a vaccine-related strategy for control.

## *SALMONELLA* VACCINE ANTIGENS

How technically feasible is the development of combination vaccines containing *Salmonella enterica* serovars Typhi, Typhimurium, Enteritidis, and Paratyphi A? These serovars are distinguished by their lipopolysaccharide O-antigen, their flagellin H-antigen, and the presence or absence of a Vi capsule [34]. Of the 4 serotypes, only *S.* Typhi is encapsulated with Vi, and so Vi-based TCVs offer no protection against the other 3 leading disease-causing serotypes.

For the non–*S.* Typhi serotypes, data from human and animal studies implicate O-antigen as the key target of protective immunity [35–37]. The O-antigens of *Salmonella* serovars share a common “backbone” consisting of repeating units of mannose, rhamnose, and galactose but are antigenically distinct due to the side chains attached to this backbone [38, 39]. *Salmonella* Enteritidis shares its O:9 antigen with *S.* Typhi, whereas *S.* Paratyphi A has an O:2 antigen and *S.* Typhimurium an O:4 or O:4,5 antigen [34].

O-antigens of gram-negative bacteria are amenable to the development of efficacious conjugate vaccines. Proof of concept was achieved 25 years ago with a *Shigella sonnei* O-antigen-rEPA vaccine developed at the US National Institutes of Health [40, 41] and, more recently, with a *Shigella flexneri* 2a-EPA vaccine developed at LimmaTech [42]. These O-antigen–based *Shigella* vaccines indicate the potential feasibility of multivalent *Salmonella* vaccines consisting of combinations of Vi, O:2, O:4, and O:9 conjugated to either a generic carrier protein, such as TT, DT of CRM_197_, or a *Salmonella*-specific protein such as flagellin [43] or SseB [44]. The O:4 and O:9 conjugate components would also likely cross-protect against other *Salmonella* serovars that share the O:4 and O:9 antigen, including *S* Dublin, *S* I:4,[5],12:i:-, and *S* Stanleyville, which are all minor causes of iNTS disease [45].

## COMBINATION *SALMONELLA* VACCINES IN DEVELOPMENT

An attractive strategy for the development of combination *Salmonella* vaccines is to base them on licensed TCVs. This approach could have an easier pathway to licensure than a completely de novo vaccine. For compatibility reasons, these vaccines would need to consist of components suitable for parenteral administration. To date, *Salmonella* combination vaccines following this approach can broadly be divided into bivalent (*S.* Typhi/Paratyphi A; Vi/O:2) enteric fever vaccines for South/Southeast Asia and trivalent (*S.* Typhi/Typhimurium/Enteritidis; Vi/O:4,5/O:9) typhoid/iNTS vaccines for Africa.

An alternative approach is to develop monovalent or bivalent vaccines lacking a typhoid component with the prospect of combining with a TCV postlicensure. As shown in Figure 1, a number of combination *Salmonella* vaccines, as well as 2 monovalent paratyphoid A vaccines, are being developed by or in partnership with manufacturers of licensed TCVs. These vaccines are mostly in early clinical development or preclinical development.

### Bivalent NTS Vaccines

#### *S.* Typhimurium/*S.* Enteritidis

Bivalent NTS vaccines are currently being developed by the GSK Vaccines Institute for Global Health (GVGH) [46, 47] and Boston Children's Hospital (BCH). The GVGH vaccine, although O-antigen based, consists of native outer membrane vesicles (NOMVs, also termed GMMA, Generalized Modules for Membrane Antigens) of *S.* Typhimurium/*S.* Enteritidis that serve as a delivery vehicle for O-antigen, in addition to presenting a multitude of other *Salmonella* outer membrane and periplasmic protein antigens to the immune system [48]. This vaccine has recently started a phase 1 trial at the University of Oxford [49].

The BCH bivalent NTS vaccine consists of O:4 and O:9 antigens of *S.* Typhimurium and *S.* Enteritidis attached to carrier protein using the proprietary multiple antigen presenting system (MAPS) technology of Affinivax (United States) [50]. This vaccine is still in preclinical development.

### Trivalent NTS and Typhoid Vaccines

#### *S.* Typhimurium/*S.* Enteritidis/*S.* Typhi

The most advanced combination *Salmonella* vaccine is a trivalent vaccine developed in partnership between the University of Maryland and Bharat Biotechnology [51]. The vaccine is based on the Bharat prequalified TCV and includes 2 NTS glycoconjugates consisting of O:4 and O:9 antigens coupled to the flagellin antigens of *S.* Typhimurium and *S.* Enteritidis respectively, in combination with Typbar TCV. This vaccine has been tested in 2 phase 1 studies in the United States and found to be safe and immunogenic with long-lasting antibody response to all components [54, 55]. The vaccine is due to be tested shortly in phase 2 studies in Africa.

Recently GVGH has started a phase 1 study in Belgium of a trivalent vaccine composed of their bivalent OMV NTS vaccine and Biological E's TYPHIBEV TCV [56]. The combination of OMV and glycoconjugate vaccine formats in the same multivalent vaccine is a novel concept. It will be interesting to compare the immunogenicity of this vaccine with the bivalent OMV NTS vaccine.

A third trivalent NTS/typhoid vaccine is being developed by SK Bioscience in partnership with the International Vaccine Institute (IVI; South Korea) [57]. Similar to the other 2 trivalent vaccines, this vaccine is based on SK Bioscience's licensed TCV, SKYTyphoid. The NTS components are both glycoconjugates consisting of O:4 and O:9 coupled to DT, the same carrier protein present in SKYTyphoid.

### Monovalent Paratyphoid A Vaccines

Although, by definition, not combination vaccines, it is helpful to be aware of 2 monovalent paratyphoid A vaccines that have been tested in the clinic. University of Maryland and Bharat Biotechnology have been developing a live attenuated *S.* Paratyphi A vaccine, CVD 1902 [58]. Following a phase 1 study [59], this has been tested in an *S.* Paratyphi A CHIM study at the University of Oxford with results pending. However, since this is an orally administered vaccine, it is incompatible for formulation with Typbar TCV. The second vaccine is an O:2-TT conjugate [60] developed by LIBP. Limited information is available on the progress of this vaccine, though it appears to be in late-stage clinical trials.

### Bivalent Enteric Fever Vaccines

#### *S.* Typhi/*S.* Paratyphi A

Combination parenteral bivalent enteric fever vaccines are in development at Biological E, SK Bioscience, Serum Institute of India, and BCH. The Biological E vaccine is being developed in partnership with GVGH and consists of a bivalent glycoconjugate vaccine based on TYPHIBEV TCV combined with an O:2-CRM_197_*S.* Paratyphi A component [61]. A phase 1 clinical trial in Belgium has recently started. Following a similar approach, SK Bioscience, in partnership with IVI, has combined its SKYTyphoid TCV with O:2-DT [62]. This vaccine is still in preclinical development.

The Serum Institute of India bivalent vaccine consists of de novo Vi-TT and O:2-DT components, neither of which have been clinically tested separately. The vaccine is currently in a phase 1 study in India. In common with its bivalent NTS vaccine, BCH's bivalent enteric fever vaccine uses MAPS technology with Vi and O:2 bound to carrier protein [63]. This vaccine is in advanced preclinical development. In contrast to other combination enteric fever vaccines, Bharat Biotech, with the University of Maryland, has chosen to combine its live attenuated *S.* Paratyphi A vaccine, CVD 1902, with a live attenuated typhoid vaccine, CVD 909, resulting in the only current oral bivalent enteric fever candidate vaccine [64]. This vaccine has yet to reach the clinic.

### Potential Quadrivalent *Salmonella* Vaccines

#### *S.* Typhimurium/*S.* Enteritidis/*S.* Typhi/*S.* Paratyphi A

Although no quadrivalent *Salmonella* vaccine is currently known to be in development, our review of existing bivalent and trivalent *Salmonella* vaccine development indicates that at least 3 vaccine development and manufacturing entities or partnerships already have the constituent components for such a vaccine. This would make the development of a quadrivalent *Salmonella* vaccine a relatively straightforward process for each vaccine developer.

With its bivalent NTS vaccine, trivalent NTS/typhoid vaccine, and bivalent enteric fever vaccine all currently in clinical trials, Biological E is the manufacturer closest to having a quadrivalent vaccine. SK Bioscience could combine its trivalent typhoid/NTS vaccine with its bivalent enteric fever vaccine to give a quadrivalent *Salmonella* vaccine consisting of 4 polysaccharide components (Vi, O:2, O:4, and O:9) conjugated to DT. Similarly, BCH could combine the 4 components from its bivalent NTS vaccine and bivalent enteric fever vaccine. Neither BCH vaccine has yet been tested in humans, and the quadrivalent vaccine would likely need manufacturing support from Affinivax (United States), which was recently acquired by GSK.

Indeed, for Bharat Biotech, the path to a quadrivalent *Salmonella* vaccine does not appear too difficult as it would just require the addition of an *S.* Paratyphi A conjugate to Bharat Biotech's trivalent NTS/typhoid vaccine. The additional *S.* Paratyphi A component would most likely be either O:2-TT or O:2-flagellin.

## KEY CONSIDERATIONS FOR *SALMONELLA* COMBINATION VACCINES

### Geography

The strongest case for the development of separate bivalent NTS and enteric fever *Salmonella* combination vaccines is epidemiological, based on the relative geographical distribution of disease caused by the 4 major *Salmonella* serovars (Figure 2). The worldwide epidemiology of *Salmonella* disease indicates that although typhoid fever is dominant in Asia, it is responsible for a minority of all *Salmonella* deaths and DALYs, particularly in Africa where iNTS disease is dominant and especially among children aged <5 years, among whom typhoid fever is the less common cause of *Salmonella* deaths and DALYs [30]. iNTS disease is largely focused in sub-Saharan Africa and uncommon in Asia, whereas *S.* Paratyphi A disease is mostly confined to South and Southeast Asia and uncommon in Africa.

At its most simplistic, the geographic distribution of disease drives the development of bivalent enteric fever vaccines for Asia and bivalent NTS vaccines for Africa. Due to the co-occurrence of *S.* Typhimurium and *S.* Enteritidis in settings where iNTS disease occurs, standalone *S.* Typhimurium and *S.* Enteritidis vaccines have never gained traction from a global health perspective. Meanwhile, typhoid fever is increasingly appreciated as a problem in Africa from recent pan-African surveillance studies, in particular the Typhoid Surveillance in Africa Program (TSAP) [65] and the Severe Typhoid in Africa (SETA) study [66], These 2 studies examined typhoid fever incidence in sites in Africa where typhoid fever has been reported to be a problem. Separately, the outbreak potential of typhoid fever makes a strong case for adding a licensed TCV to bivalent NTS vaccine combinations.

The parallel argument of adding NTS components to a bivalent enteric fever vaccine for Asia is not as strong. However, dNTS disease is universal, and, although a lesser cause of mortality according to GBD 2019 than iNTS disease, indicates the potential utility in Asia of a quadrivalent *Salmonella* vaccine that includes the 2 key global NTS serovars, as well as *S.* Typhi and *S.* Paratyphi A components. Would a quadrivalent vaccine also have value in Africa? At present, low incidence of paratyphoid A fever in Africa argues against this, though epidemiology is, by nature, fluid. Furthermore, a single combination vaccine that could be used in all geographies, similar to licensed multivalent pneumococcal and meningococcal conjugate vaccines, and quadrivalent *Shigella* vaccines in clinical development [67], is attractive.

### Age of Administration

Age of administration of combination *Salmonella* vaccines requires careful consideration. TCVs introduced nationally are currently administered with measles-containing vaccines at the WHO 9-month or 15-month Expanded Programme on Immunization (EPI) time point. This age is compatible with bivalent enteric fever vaccines in development, as paratyphoid A is uncommon in children aged <9 months.

A potential drawback to a trivalent NTS/typhoid vaccine is the different age profiles of iNTS disease and typhoid fever. iNTS disease is common in infancy after the waning of maternal antibody [68], peaking at around 1 year of age, whereas typhoid fever is more common in later childhood. Therefore, the 9-month time point is too late for use of an NTS-containing *Salmonella* vaccine. Licensure of TCVs down to 6 months of age permits a change in administration time to this younger age. No EPI vaccines are currently administered at this time, though the situation could change in Africa with the introduction of the RTS,S/AS01 and R21 malaria vaccines. Another option would be to use combination *Salmonella* vaccines containing NTS and typhoid components at the 14-week EPI timepoint. A drawback is the crowded nature of the early EPI schedule, making introduction of another vaccine difficult at this time point.

### Dose Scheduling

A surprising finding of the TCVs has been their efficacy after a single dose, even in young children [16–18]. Though a booster dose may be necessary after some years, it is now clear that a single-dose primary schedule of TCV confers excellent levels of protection for at least 3 years and down to the youngest age groups. Ideally *Salmonella* combination vaccines will also only require a 1-dose primary schedule. Should additional doses be needed to confer protective immunity against the 3 nontyphoid components, this will increase the delivery cost of using *Salmonella* combination vaccines over TCV alone.

### Cost

Cost is a major consideration for all vaccines, particularly those targeted primarily for use in LMICs, and increasing vaccine valency adds complexity in relation to formulation and quality control, which can increase costs [69]. However, there are potential cost savings associated with multivalent vaccines compared with monovalent vaccines. For example, with *Haemophilus influenzae* b (Hib)–containing vaccines, the cost of adding Hib as a component of a multivalent vaccine was found to be less than the cost of a monovalent Hib vaccine [70]. Furthermore, the need for high-valency pneumococcal vaccines has driven innovation in vaccine manufacturing technologies to help maintain affordability. Indeed, Gavi's Advanced Market Commitment enabled Pneumosil, a 10-valent pneumococcal conjugate vaccine (Serum Institute of India), to be made available to LMICs at $2 per dose [71].

Moreover, delivery costs, cold chain storage costs, and biohazard disposal costs of multivalent vaccines are reduced compared with administering multiple monovalent vaccines [70]. A particularly attractive scenario for *Salmonella* vaccines is the replacement of nationally introduced monovalent TCVs with combination *Salmonella* vaccines. Provided these are administered at EPI vaccination timepoints and only a single dose is required for protection (see above), this “vaccine swap” scenario would potentially negate any new delivery cost for *Salmonella* combination vaccines over monovalent TCV.

### Speed of Development

Vaccine development normally takes many years. Development of multiple combination vaccines tailored to different geographies potentially adds time to the broad development of *Salmonella* combination vaccines. This factor provides a strong reason to focus on the development of a quadrivalent combination vaccine that can be used in all LMIC geographies.

## VACCINES FOR OTHER PATHOGENS

Multivalent vaccines for other bacterial pathogens can serve as valuable examples when considering options for combination *Salmonella* vaccines. Pneumococcal conjugate vaccines provide the best examples of magnitude of valency not being an insurmountable obstacle. The 7-valent Prevenar conjugate vaccine was replaced by the 13-valent Prevenar conjugate vaccine, and newer vaccines of 20 or more valencies are in clinical development. Importantly, the same pneumococcal conjugate vaccines are used across multiple geographies, even though the geographic burden of each serotype is highly variable.

The quadrivalent meningococcal conjugate vaccines, consisting of meningococcal group A, C, W, and Y components, provide the best exemplar for development of *Salmonella* quadrivalent combination vaccines [72]. Even though meningococcus group A rarely causes disease in high-income countries, this has not proved an obstacle to the introduction and use of quadrivalent meningococcal vaccines in many high-income countries. In the United Kingdom, meningococcal ACWY conjugate vaccine rapidly replaced the monovalent meningococcal C vaccine following a rise in cases of meningitis caused by group W [73]. These examples show that the variable geographic burden of disease caused by vaccine components has not historically been a deterrent for broad use.

In relation to similarity of pathogen, *Shigella* conjugate vaccines are helpful to consider. Here, the large number of serotypes that cause disease has led to careful consideration about which serotypes to include in a *Shigella* vaccine. Though there are no licensed *Shigella* vaccines to date, the most advanced *Shigella* vaccines share a quadrivalent format consisting of *Shigella flexneri* 2a, 3a, and either 6 or 1b, together with *Shigella sonnei*, based on global epidemiological data [74]. There has been no move to develop different *Shigella* combination vaccines based on regional differences in disease-causing serotype distribution, an approach with potential benefits in relation to simplicity, cost, and time to licensure.

## PATHWAYS TO LICENSURE AND INTRODUCTION

Currently, 5 *Salmonella* combination vaccines have reached phase 1 clinical trials and none has yet advanced beyond this point. Pathways to licensure for such vaccines are a current topic of interest for the WHO Product Development Vaccine Advisory Committee [75]. Questions remain as to whether combination vaccines will require full field efficacy studies for licensure or could be licensed by other means including CHIM studies and immunobridging to TCV efficacy studies.

The relatively low incidence of paratyphoid A fever has led many to question whether a field efficacy study for a paratyphoid A vaccine, or the paratyphoid A component of a combination vaccine, is feasible. In the context of a bivalent enteric fever vaccine, the development of a paratyphoid A CHIM at the University of Oxford [76] provides a means by which efficacy could be demonstrated for the paratyphoid A component without a field efficacy trial.

For vaccines containing NTS components, the high incidence of iNTS disease in many settings in Africa makes a field efficacy study feasible and therefore likely to be required for licensure. An NTS CHIM is currently in development at Imperial College, London, and could be used to derisk the development of such a vaccine by providing an early indication of efficacy [77].

In relation to introduction of *Salmonella* combination vaccines following licensure, as previously mentioned, where TCV has already been introduced, direct replacement with a combination vaccine is a possible scenario.

## CONCLUSIONS

Efficacious TCVs have recently been introduced nationally in several LMICs. However, with typhoid representing less than half the global burden of *Salmonella* disease, there is a strong case to develop combination *Salmonella* vaccines containing the other main disease-causing serovars of *Salmonella enterica*: *S.* Typhimurium, *S.* Enteritidis, and *S.* Paratyphi A. Trivalent typhoid/NTS vaccines would be particularly suitable for Africa, and bivalent enteric fever vaccines for South/Southeast Asia. Alternatively, and perhaps move attractively, quadrivalent *Salmonella* vaccines covering all 4 key serotypes could be used across the range of LMIC geographies.

## Notes

***Financial support.*** This work received no external financial support.

***Supplement sponsorship.*** This article appears as part of the supplement “Charting the Course to Meet the Challenges Ahead: Research and Developments on Typhoid and Other Invasive Salmonelloses” sponsored by the Coalition against Typhoid Secretariat, housed at the Sabin Vaccine Institute in Washington, DC and made possible by a grant from the Bill & Melinda Gates Foundation.
